# Polymeric micelle hydrogel of *Resina Draconis* enables mechanistically targeted protection against skin photoaging

**DOI:** 10.1186/s13020-026-01344-w

**Published:** 2026-02-16

**Authors:** Mengzhu Liu, Yuqing Huang, Wanyang Sun, Yuqi Wang, Xiu Yu, Huichao Xie, Pingtian Ding, Yuanyuan Xie, Keda Zhang

**Affiliations:** 1 School of Pharmacy, Shenzhen University Medical School, Shenzhen University, Shenzhen, 518055 China; 2 College of Pharmacy, Shenzhen Technology University, Shenzhen, 518118 China; 3 School of Chinese Materia Medica, Guangdong Pharmaceutical University, Guangzhou, 510006 China; 4 State Key Laboratory of Bioactive Molecules and Druggability Assessment/Guangdong Engineering Research Center of Traditional Chinese Medicine & Disease Susceptibility/Guangdong Engineering Research Center of Traditional Chinese Medicine & Health Products/Institute of Traditional Chinese Medicine and Natural Products, College of Pharmacy, Jinan University, Guangzhou, 510632 China; 5 School of Pharmacy, Shenyang Pharmaceutical University, Shenyang, 110016 China; 6 Department of Pulmonary and Critical Care Medicine, Shenzhen Key Laboratory of Respiratory Diseases, Shenzhen Clinical Research Center for Respiratory Disease, Shenzhen Institute of Respiratory Diseases, Shenzhen People’s Hospital (The First Affiliated Hospital, Southern University of Science and Technology, The Second Clinical Medical College, Jinan University), Shenzhen, 518020 China

**Keywords:** *Resina Draconis*, Polymeric micelle, Photoaging, Topical delivery, Metabolomics

## Abstract

*Resina Draconis* (*RD*), a traditional Chinese medicinal resin renowned for its potent antioxidant, anti-inflammatory, and tissue-repairing properties, holds promise for combating skin photoaging. However, its therapeutic translation has been hindered by the poor solubility and limited bioavailability of its principal bioactive constituents. In this study, we developed an *RD*-loaded polymeric micelle (RDPM) hydrogel to overcome these formulation challenges and evaluated its protective efficacy against UV-induced skin aging. The micellar system, composed of poloxamer surfactants and embedded within a carbomer hydrogel matrix, achieved high encapsulation efficiencies (~ 80–90%) for five representative bioactive constituents (loureirin A, loureirin B, 7,4′-dihydroxyflavone, resveratrol, and pterostilbene). The micellar hydrogel exhibited diffusion-governed release (~ 80% within 24 h), consistent with Higuchi kinetics. In vitro permeation studies confirmed efficient penetration of these actives across the stratum corneum and deposition within the viable epidermis and dermis, the target compartments for anti-photoaging action. In UV-irradiated mice, topical RDPM hydrogel treatment markedly ameliorated wrinkle formation and histopathological alterations, including epidermal hyperplasia, dermal–epidermal junction flattening, collagen loss, and elastotic degeneration. Untargeted metabolomic profiling further showed a treatment-associated normalization of UV-disrupted metabolic networks, consistent with improvement of redox balance, restoration of arginine-NO signaling, and preservation of mitochondrial function. A Draize skin irritation assay classified the formulation as non-irritating. Collectively, this study establishes RDPM hydrogel as a rationally designed topical delivery system that amplifies the pharmacodynamic benefits of *RD*, offering a mechanistically supported intervention for photoaging within the paradigm of modern Chinese medicinal pharmaceutics.

**Graphical Abstract:**

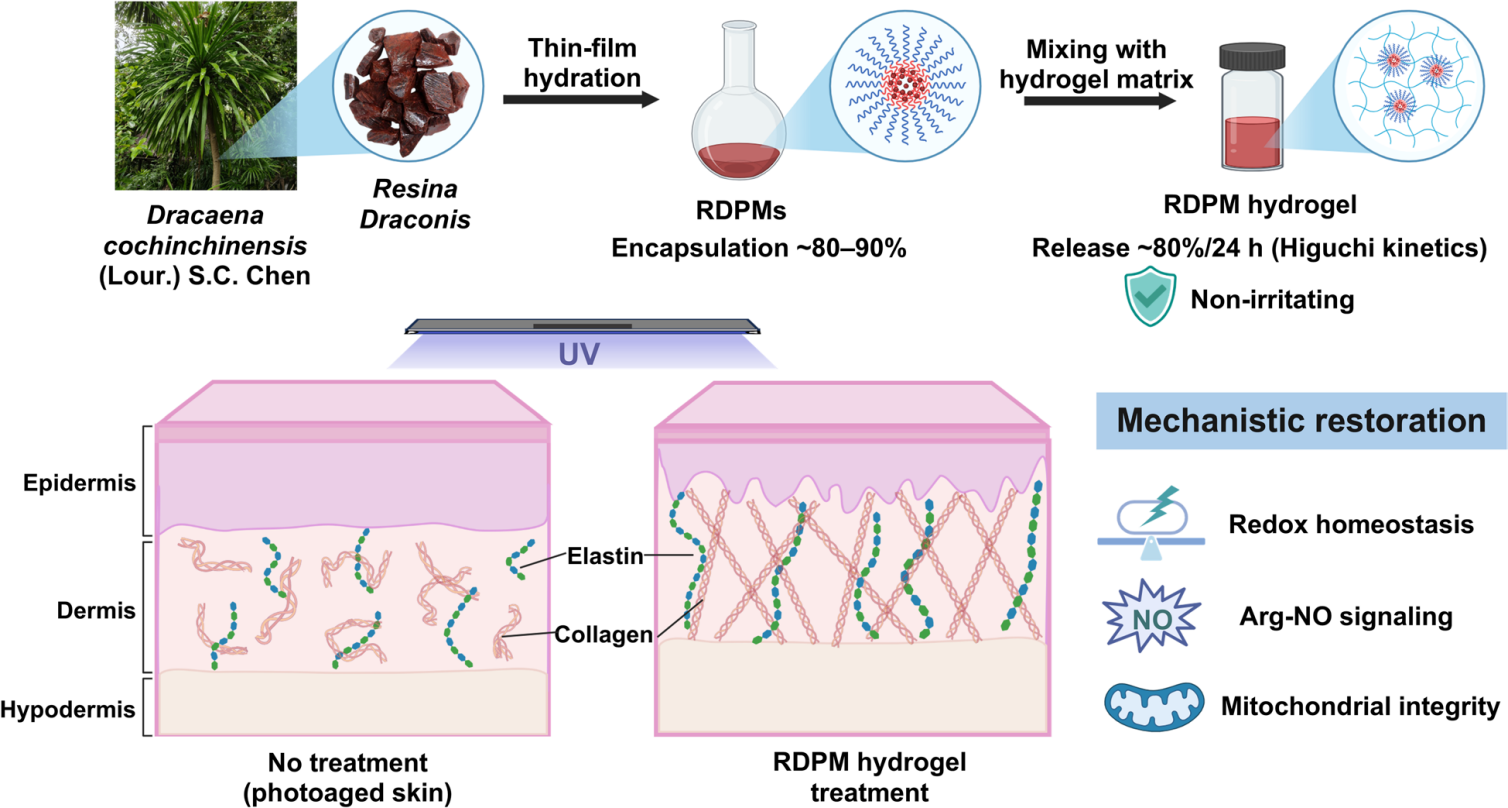

**Supplementary Information:**

The online version contains supplementary material available at 10.1186/s13020-026-01344-w.

## Introduction

Skin photoaging refers to the premature aging of the skin caused by chronic exposure to ultraviolet (UV) radiation, primarily from sunlight. Unlike intrinsic aging, which is genetically programmed, photoaging results from cumulative extrinsic environmental insults that lead to characteristic clinical and histological manifestations, including coarse wrinkles, dyspigmentation, loss of elasticity, and surface roughness [1]. At the molecular level, UV radiation induces the excessive generation of reactive oxygen species (ROS), resulting in oxidative stress, DNA damage, and the activation of multiple signaling cascades such as the mitogen-activated protein kinase (MAPK) and nuclear factor kappa B (NF-κB) pathways [2]. These pathways upregulate matrix metalloproteinases (MMPs) that degrade collagen and other extracellular matrix (ECM) components while concurrently suppressing collagen synthesis via inhibition of transforming growth factor-β (TGF-β) signaling [3, 4]. Persistent inflammation and mitochondrial dysfunction further aggravate these processes, leading to structural deterioration and functional decline of the skin [5, 6]. A deeper understanding of these molecular mechanisms provides a foundation for developing targeted therapeutic interventions to prevent or reverse photoaging.

In recent years, natural products with potent antioxidant and anti-inflammatory properties have attracted growing interest for their potential roles in mitigating skin photoaging [7, 8]. *Resina Draconis* (*RD*; also known as Long Xue Jie), a red resin extracted from stems of *Dracaena cochinchinensis* (Lour.) S.C. Chen, has drawn attention due to its diverse bioactive constituents and pharmacological activities [9]. Chemically, *RD* contains abundant flavonoids and stilbenoids, which exhibit well-documented free-radical scavenging, anti-inflammatory, and tissue-repairing properties [10, 11]. These compounds suppress oxidative stress by activating the nuclear factor erythroid 2-related factor 2/heme oxygenase-1 (Nrf2/HO-1) antioxidant pathway, reducing lipid peroxidation, and enhancing the activities of cellular defense enzymes such as superoxide dismutase (SOD) and glutathione peroxidase (GSH-Px) [12–15]. In addition, *RD* and its constituents inhibit NF-κB and MAPK/activator protein-1(AP-1) signaling cascades, leading to downregulation of pro-inflammatory cytokines downregulation of pro-inflammatory cytokines—tumor necrosis factor-α (TNF-α), interleukin-1β (IL-1β), and IL-6—and inhibition of MMP-1 and MMP-3, key mediators of collagen degradation and ECM disorganization during photoaging [16, 17]. Furthermore, *RD* promotes fibroblast proliferation and collagen biosynthesis through activation of the TGF-β/Smad signaling cascade, contributing to dermal structural restoration following photodamage [18]. Together, the multifunctional activities highlight the potential of *RD* as a multitarget natural therapeutic candidate for the prevention and treatment of UV-induced skin photoaging.

Despite *RD*’s promising pharmacological potential, the extremely poor solubility of its chemically diverse bioactive constituents (e.g., flavonoids and stilbenoids) in both aqueous and lipid media severely compromises dissolution and bioavailability, thereby posing substantial challenges for formulation development and clinical translation [19, 20]. In our preliminary screening, widely used topical delivery systems, such as conventional micelles, liposomes, and microemulsions, failed to achieve meaningful enhancement of *RD* solubilization (see Fig. S1), highlighting the need for an alternative formulation strategy. In this study, we developed an *RD*-loaded polymeric micelle (RDPM) hydrogel, in which *RD* was first efficiently encapsulated into polymeric micelles to markedly enhance its apparent aqueous solubility and dispersion stability, and the micelles were subsequently embedded in a hydrogel matrix to enable practical topical administration and effective release of *RD* constituents. We further evaluated the protective efficacy of the RDPM hydrogel against UV-induced skin photoaging in hairless mice and investigated its mechanistic basis through comprehensive untargeted metabolomic profiling. Collectively, this study advances the topical application of *RD* for the protection against skin photoaging.

## Materials and methods

### Chemicals

*RD* powder was obtained from Banna Pharmaceutical (Jinghong, China). To ensure the chemical consistency and quality control of the raw material used for subsequent formulation studies, the contents of five representative bioactive constituents, including loureirin A (LA), loureirin B (LB), 7,4′-dihydroxyflavone (DHF), pterostilbene (PTE), and resveratrol (RES), were simultaneously quantified using liquid chromatography coupled with triple quadrupole tandem mass spectrometry (LC-QqQ-MS/MS) under optimized multiple reaction monitoring (MRM) conditions (Table S1). The analytical procedures are described in detail below. Pharmaceutical-grade Poloxamer 188 and triethanolamine were provided by BASF (Ludwigshafen, Germany). Carbomer 940 (pharmaceutical grade) was purchased from Lubrizol (Wickliffe, OH, USA), and glycerin (pharmaceutical grade) was obtained from Yipusheng Pharmaceutical (Ji’an, China). Reference standards of LA, LB, DHF, PTE, and RES (≥ 98% purity, HPLC-verified) were purchased from Yuanye Bio-Technology (Shanghai, China). All other reagents and solvents were of analytical grade or higher.

### Animals

Female specific pathogen-free (SPF) SKH-1 hairless mice (16–24 g, 4–6 weeks old) were obtained from Beijing Vitalstar Biotechnology Co., Ltd. (Beijing, China), and male SPF guinea pigs (300–350 g, 4–6 weeks old) were sourced from Guangdong Medical Laboratory Animal Center (Foshan, China). The animals were allowed to acclimate for one week under laboratory conditions. The room temperature and relative humidity were maintained at 24–26 °C and 60–80%, respectively, with a 12-h light/dark cycle. Throughout the study, the mice had unrestricted access to food and water. All experimental procedures were approved by the Animal Ethics Committee of Shenzhen Technology University (SZTUDWLL2024047) and were conducted in strict accordance with the Guide for the Care and Use of Laboratory Animals of China.

### Preparation of *RD*-loaded polymeric micelles

Poloxamer 188 (1.0 g) and *RD* powder (100 mg) were placed into a 250 mL round-bottom flask, followed by the addition of 50 mL of methanol. The mixture was sonicated until completely dissolved. The solvent was then gradually removed under reduced pressure at 50℃ using a rotary evaporator, leading to the formation of a thin film on the inner wall of the flask. Subsequently, an appropriate volume of ultrapure water is added, followed by ultrasonic hydration at 35 ℃ for 45 min with vigorous agitation. The resulting dispersion was centrifuged, and the supernatant containing the *RD*-loaded polymeric micelles (RDPMs) was collected. The particle size distribution and zeta potential of the RDPMs were determined using a Zetasizer Pro (Malvern Panalytical, Malvern, UK). The morphology and size of the micelles were observed by transmission electron microscopy (TEM; JEM-1400; JEOL, Tokyo, Japan). The encapsulation efficiency and drug loading of five representative bioactive constituents (LA, LB, DHF, PTE, and RES) were quantified using an ultrafiltration method. Briefly, a defined volume of the RDPM dispersion was placed in an ultrafiltration centrifuge tube (molecular weight cut-off 10 kDa) and centrifuged at 8,000 rpm for 15 min to separate unencapsulated drug from the micellar fraction. The concentrations of free and total drug were quantified by LC-Q-Orbitrap MS/MS, and the encapsulation efficiency (EE%) and drug loading (DL%) were calculated according to the following equations:

1 $$EE \left(\%\right)= \left(\frac{{W}_{encapsulated}}{{W}_{total}}\right)\times 100$$

2 $$DL \left(\%\right)= \left(\frac{{W}_{encapsulated}}{{W}_{micelles}}\right)\times 100$$

where ${W}_{encapsulated}$ is the amount of drug entrapped in the micelles, ${W}_{total}$ is the total amount of drug used, and ${W}_{micelles}$ is the total weight of the micelles.

### Hydrogel preparation

Carbomer 940 powder (0.04 g) was uniformly dispersed in an appropriate amount of ultrapure water and allowed to stand overnight to ensure complete swelling. Triethanolamine was then added to adjust the pH to 6.0‒7.0, and the mixture was stirred to form the hydrogel matrix. Subsequently, the previously prepared solution of RDPM and 1.0 g of glycerol were added, and ultrapure water was supplemented to achieve a total mass of 10 g. The mixture was thoroughly stirred to obtain RDPM hydrogel (pH ~ 6.5). The microstructure of the lyophilized hydrogel was examined using scanning electron microscopy (SEM; Quattro S, Thermo Fisher Scientific, Waltham, MA, USA). In addition, the morphology of micelles within the hydrogel (after dilution with water) was observed by TEM to assess the potential influence of the hydrogel matrix on micellar integrity.

### In vitro release test

An automated transdermal diffusion system (RT800, Raytor, Shenzhen, China) equipped with Franz diffusion cells (effective diffusion area: 2.00 cm^2^; receptor chamber volume: 30 mL) was employed to evaluate the in vitro drug release behavior of the RDPM hydrogel. To maintain sink conditions, a 50% (v/v) ethanol–water solution was used as the receptor medium. The receptor chamber was maintained at 37 °C using a dry-heat module, and continuously stirred with a magnetic bar. A dialysis membrane (molecular weight cut-off: 12–14 kDa, Medicell, London, UK) was mounted between the donor and receptor compartments of the diffusion cell. After a 60-min equilibration period, air bubbles in the receptor medium were carefully removed through the sampling port. Subsequently, a 300 mg aliquot of the RDPM hydrogel was applied evenly to the donor chamber and sealed with Parafilm to prevent evaporation. At predetermined time intervals, 1.0 mL of receptor solution was withdrawn and immediately replenished with an equal volume of fresh receptor medium. Each experiment was conducted in six replicates (n = 6). The collected samples were filtered through 0.22 μm syringe filters, and the concentrations of LA, LB, DHF, PTE, and RES were quantitatively determined using LC-QqQ-MS/MS.

### In vitro skin permeation study

Full-thickness porcine skin was obtained from Shenzhen Scientific & Excellent Medical Testing Co., Ltd. (Shenzhen, China). After the removal of subcutaneous fat and hair shafts, the skin was cleaned with phosphate-buffered saline (PBS) and stored at −20 °C. Prior to the experiments, skin disks (⌀ 25 mm) were punched from the frozen skin and thawed at room temperature. The structural integrity of the skin disks was confirmed using a transdermal electrical resistance (TER) test, as previously reported [21].

The in vitro skin permeation study was performed using the same Franz diffusion system as described above. The porcine skin disks were mounted onto the diffusion cells with the stratum corneum (SC) facing upward toward the donor chamber. The receptor chambers were filled with a 50% (v/v) ethanol-PBS (pH 7.4) solution to serve as the receptor medium and equilibrated for 60 min. Subsequently, a 150 mg aliquot of RDPM hydrogel was evenly applied to the skin surface. At predetermined time intervals, 3 mL of receptor medium was withdrawn and immediately replaced with an equal volume of fresh receptor solution. Three independent replicates (n = 3) were performed. The collected samples were vacuum-concentrated and redissolved in 300 μL of methanol, followed by centrifugation at 12,000 rpm for 10 min. The resulting supernatant was collected for the quantitative analysis of LA, LB, DHF, PTE, and RES using LC-QqQ-MS/MS.

### Skin deposition analysis

Following the 24-h in vitro skin permeation study, the skin disks were carefully removed from the diffusion cells, thoroughly rinsed with PBS, and gently blotted dry with filter paper. The SC was collected using a tape-stripping method. Briefly, each skin disk was placed onto a home-made holder and covered with a Teflon membrane featuring a pre-cut circular window (15 mm in diameter). A 2 cm-long piece of adhesive tape was applied to the exposed skin area, and uniform pressure was applied using a 2 kg weight for 10 s. The tape was then swiftly removed with tweezers. This process was repeated ten times, and all collected tapes were transferred into glass vials containing 4 mL of 50% (v/v) methanol–water solution, ensuring complete immersion. The viable epidermis was then carefully scraped off with a scalpel and transferred into 2 mL vials containing 1 mL of 50% (v/v) methanol–water solution. The remaining dermis was finely minced using a scalpel and soaked in 1 mL of 50% (v/v) methanol–water solution. All vials were agitated overnight at room temperature to facilitate complete extraction of the bioactive compounds. After extraction, all samples were filtered through 0.22 μm syringe filters, and the resulting filtrates were analyzed by LC-QqQ-MS/MS to quantitatively determine the deposition profiles of the five *RD* constituents (LA, LB, DHF, PTE, and RES) across different skin layers.

To verify extraction completeness for skin deposition quantification, a sequential re-extraction procedure (mass-balance) was performed, as described in the supplementary materials. The first extraction recovered ≥ 97.50% of the total amount obtained across the first to third extractions for each constituent within each skin layer (Table S2), suggesting high extraction efficiency and minimizing the likelihood of appreciable underestimation of skin deposition.

### LC-QqQ-MS/MS analysis

Quantitative determination of the *RD* constituents was performed using an Agilent 1290 Infinity II liquid chromatography system (Agilent Technologies, Waldbronn, Germany) coupled with an Agilent 6475 LC/TQ triple quadrupole mass spectrometer (Agilent Technologies, Singapore). Chromatographic separation was achieved on a Waters Acquity UPLC^®^ HSS T3 column (1.8 μm, 2.1 × 50 mm; Waters, Wexford, Ireland) equipped with a VanGuard™ pre-column (1.8 μm, 2.1 × 5 mm; Waters, Wexford, Ireland). The column temperature was maintained at 30 °C, with a flow rate of 0.4 mL/min, and the injection volume was 2 μL. The mobile phase consisted of 0.1% formic acid in water (A) and 0.1% formic acid in acetonitrile (B), employing the following gradient elution program: 0–2 min, 25% B; 2–3 min, 25–45% B; 3–6 min, 45% B; 6–7 min, 45–100% B; 7–9 min, 100% B; 9–9.1 min, 100–5% B; and 9.1–11 min, 25% B.

Mass spectrometric detection was carried out in negative ionization mode using MRM. The optimized MS parameters were: capillary voltage, −3.5 kV; nozzle voltage, −500 V; drying gas temperature, 300 °C; drying gas flow, 5 L/min; nebulizer pressure, 35 psi; sheath gas temperature, 250 °C; and sheath gas flow, 11 L/min. Quantification was based on specific MRM transitions for each analyte as follows: LA, *m/z* 285.3 → 134.0; LB, *m/z* 315.3 → 133.9; DHF, *m/z* 253.2 → 117.0; RES, *m/z* 227.1 → 143.1; and PTE, *m/z* 255.1 → 240.0.

### Photoaging mouse model and treatment

Female SKH-1 hairless mice were randomly assigned to five groups (n = 8 per group): normal, model, blank hydrogel, low-dose, and high-dose groups. Except for the normal group, all mice underwent photoaging induction by UV irradiation for 15 weeks using a combination of three UVA lamps (UVA-340) and three UVB lamps (UVB-313EL) obtained from Q-Lab (Cleveland, OH, USA). Mice were housed in flat stainless-steel cages and exposed to UV irradiation three times per week (Monday, Wednesday, and Friday). The total UV irradiance was monitored using a digital UV meter (SDR2040, Speedre, Shenzhen, China), and the minimal erythema dose (MED) on dorsal skin was approximately 45 mJ/cm^2^. The irradiation dose was set at 1 MED during the first week, increased by 1 MED per week until reaching 4 MED by the fourth week, and maintained at this level for the remaining exposure period.

For topical treatment, 160 mg of RDPM hydrogel containing 0.25% (low dose) or 1.0% (high dose) *RD* was applied once daily to the dorsal skin throughout the irradiation period. The blank hydrogel group received an equivalent amount of drug-free polymeric micelle hydrogel, while the normal and model groups remained untreated. Body weights were recorded weekly to monitor systemic health. Two days after the final UV exposure, mice were anesthetized with isoflurane, and dorsal skin photographs were taken. Silicone replicas of the dorsal skin were prepared using SILFLO^®^ silicone impression material (Flexico, Colchester, UK), and wrinkle formation was imaged using the Visioline^®^ VL650 system (Courage & Khazaka, Cologne, Germany). The mice were then euthanized with CO_2_ asphyxiation, and dorsal skin samples were excised.

For histopathological examinations and quantitative analyses, three mice per group were randomly selected prior to tissue processing, due to the labor-intensive workflow. Small tissue sections from these animals were fixed and processed for histopathological examination, including hematoxylin–eosin (H&E) staining for evaluating general morphology and epidermal thickness, Masson’s trichrome staining for assessing total collagen content, and Gomori’s aldehyde fuchsin staining for determining elastic fiber integrity. Slides were scanned using an Aperio GT450 whole-slide scanner (Leica Biosystems, Danvers, MA, USA), and histological parameters were quantified using ImageJ software (v1.51a, NIH, USA). The remaining skin tissue samples were snap-frozen and stored for subsequent metabolomic analysis.

### Metabolomics analysis

#### Sample pre-processing

Skin tissue samples (100 mg each) were collected from individual mice and placed into centrifuge tubes containing 1 mL of pre-cold methanol. The samples were homogenized using a high-throughput tissue homogenizer (SRT-24, SAIERTE, Shenzhen, China) set to a frequency of 65 Hz for 6 min, followed by centrifugation at 12,000 rpm for 10 min at 4 °C. Next, 500 μL of the supernatant was transferred to a 1.5 mL centrifuge tube and evaporated using a vacuum concentrator (Mars CV610, FTFDS, Hangzhou, China) at 4 °C and 1400 rpm. The obtained samples were reconstituted in 100 μL of methanol for subsequent LC-Q-Orbitrap MS/MS analysis.

#### LC-Q-Orbitrap MS/MS analysis

Chromatographic analysis was conducted using a Dionex Ultimate 3000 UHPLC system (Thermo Fisher Scientific, Sunnyvale, CA, USA) equipped with a quaternary pump, an online degasser, an autosampler, and a column temperature controller. Separation of samples was achieved on a Waters Acquity UPLC® HSS T3 column (1.8 μm, 2.1 × 100 mm; Waters, Wexford, Ireland). The mobile phase consisted of 0.1% (v/v) formic acid in water (A) and acetonitrile (B), with gradient elution programmed as follows: 0–0.5 min, 5% B; 0.5–6.5 min, 5–50% B; 6.5–10.5 min, 50–60% B; 10.5–13.5 min, 60–70% B; 13.5–17.5 min, 70–95% B; 17.5–20 min, 95% B; 20–20.5 min, 95–5% B; and 20.5–23 min, 5% B. The column temperature was maintained at 35 °C, and the sample chamber was kept at 4 °C. The injection volume was 2 μL, and the flow rate was 0.4 mL/min.

The UHPLC system was coupled to a Q-Exactive hybrid quadrupole-Orbitrap mass spectrometer (Thermo Fisher Scientific, San Jose, CA, USA) equipped with a heated electrospray ionization (HESI) source. HESI-MS analysis was performed in both positive and negative ionization modes using a full scan over an m/z range of 100–1500, with a resolving power of 70000 full width at half maximum (FWHM) at *m/z* 200. Instrument settings included a spray voltage of + 3.5 kV/−3.0 kV, a sheath gas pressure of 45 arbitrary units, an auxiliary gas pressure of 10 arbitrary units, a heater temperature of 320 °C, and a capillary temperature of 350 °C. MS/MS data were acquired using a stepped normalized collision energy of 15–35% in data-dependent acquisition mode, where the most intense precursor ions from the full scan spectra were selected for fragmentation in each cycle. The resolving power for MS/MS scans was set to 17500 FWHM at *m/z* 200. For both full MS and MS/MS events, the automatic gain control (AGC) target was set to 1 × 10^5^ ions. The maximum injection times were set to 100 ms for full MS and 50 ms for MS/MS.

#### Data processing and analysis

Raw LC–MS/MS data were processed using Compound Discoverer 3.3 (Thermo Fisher Scientific) for peak extraction, background correction, deconvolution, alignment, integration, and normalization of ion intensities. The resulting data matrix was subsequently imported into MetaboAnalyst 6.0 (https://www.metaboanalyst.ca/) for multivariate statistical analysis, including principal component analysis (PCA), partial least squares discriminant analysis (PLS-DA) and orthogonal partial least squares discriminant analysis (OPLS-DA). Differential metabolites between the model and control groups were identified based on variable importance in projection (VIP) scores > 1 from the OPLS-DA model and two-tailed Student’s *t*-tests (p < 0.05). Among these, metabolites associated with the protective effects of the RDPM hydrogel were determined by comparative analysis between the model and treatment groups, where expression trends in the model group were reversed or ameliorated upon treatment. Metabolite annotation and putative identification were performed using the Human Metabolome Database (HMDB) (http://www.hmdb.ca/) and the Kyoto Encyclopedia of Genes and Genomes (KEGG) (http://www.genome.jp/kegg/). Pathway enrichment and topology analyses were subsequently conducted on the MetaboAnalyst 6.0 platform. Pathways with an impact value > 0.1 and p < 0.05 were considered to be the most significantly perturbed and biologically relevant.

### Skin irritation test

A skin irritation test was conducted using four guinea pigs. The fur on the dorsal region of the guinea pigs was shaved using an electric shaver, and the skin was examined 24 h later for any signs of erythema, edema, or other damage. Animals exhibiting damaged skin were excluded from the test. A total of 250 mg of RDPM hydrogel was applied to the right side of the guinea pigs' backs, while the left side was left untreated as a control. Photographs of the treated and control skin areas were taken at 1 h, 24 h, 48 h, and 72 h post-application. Observations of erythema and edema were recorded, and skin irritation was scored based on the Draize skin assessment criteria (Table S3 and S4). In addition, the same procedure was performed on another group of guinea pigs, which were sacrificed using CO_2_ gas at 24 h, 48 h, 72 h, and 240 h following the topical application of RDPM hydrogel. Skin samples were collected and subjected to histopathological examination using H&E staining. Skin sections were imaged using the Aperio GT450 digital slide scanner, and the number of skin cells within the area of interest (450 μm × 150 μm, encompassing only the dermis layer) was quantified using the ImageJ software.

### Statistical analysis

All statistical analyses were performed using GraphPad Prism 10.0 (GraphPad Software, San Diego, CA, USA). Data are expressed as mean ± standard deviation (SD). A two-tailed unpaired Student’s *t*-test was used for comparisons between two groups, while one-way analysis of variance (ANOVA) was used for comparisons among multiple groups. Significance levels were denoted as follows: ^*^p < 0.05, ^**^p < 0.01 ^***^p < 0.001, and ^****^p < 0.0001.

## Results

### Characterization of RDPM hydrogel

**Fig. 1:**
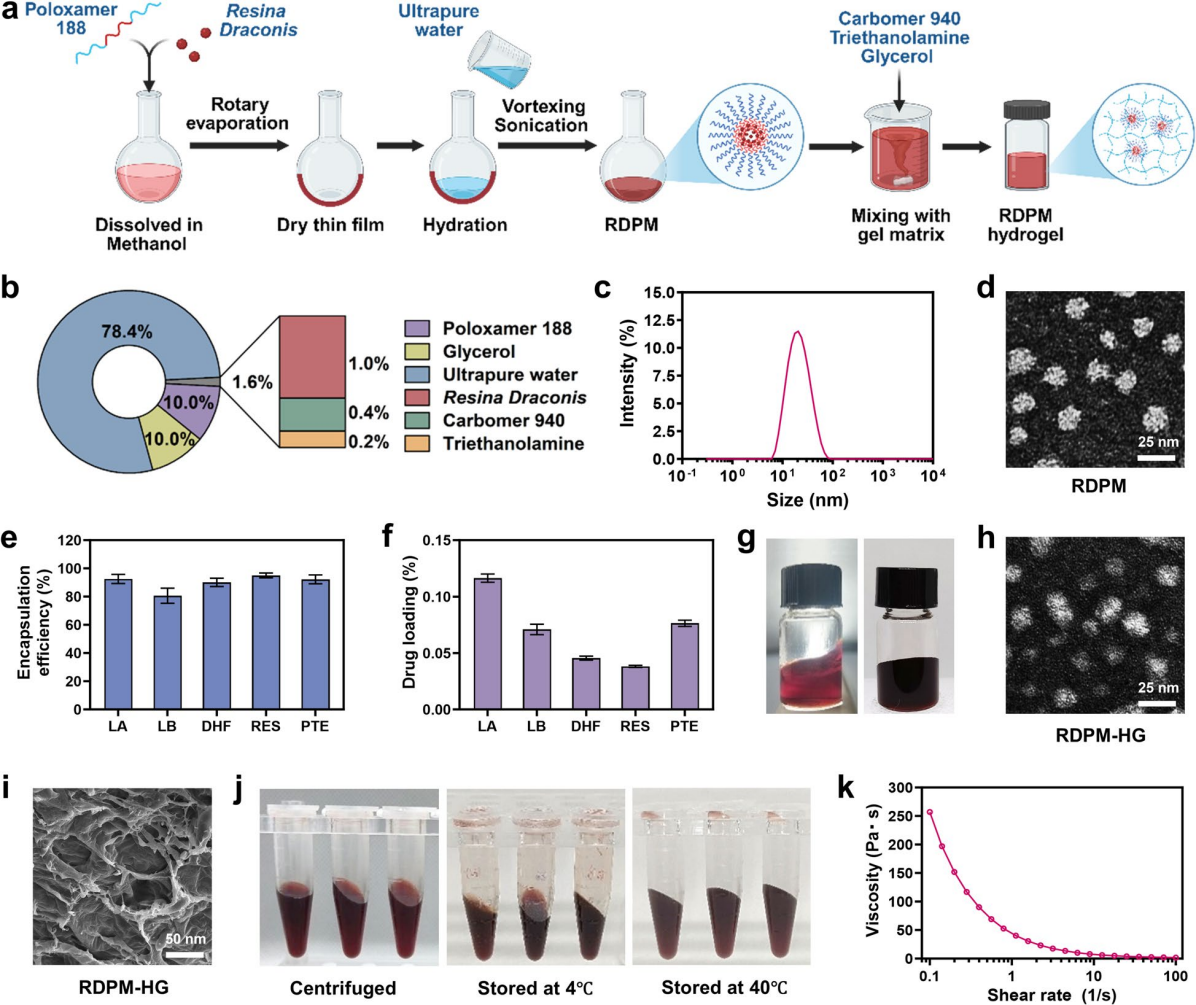
Preparation and characterization of the RDPM hydrogel. **a** Schematic illustration of the preparation process of the RDPM hydrogel. **b** Formulation composition of the RDPM hydrogel. **c** Particle size distribution profile of RDPMs. **d** TEM images of RDPMs. **e** Encapsulation efficiencies (%) of five representative bioactive constituents (LA, LB, DHF, RES, and PTE) of *RD* in RDPMs. **f** Drug loading (%) of these five active constituents in the RDPMs. **g** Macroscopic appearance of the RDPM hydrogel under incandescent light (left) and natural light (right). **h** TEM image of RDPM hydrogel after dilution with water. **i** SEM image showing the microstructure of the RDPM hydrogel. **j** Stability of the RDPM hydrogel after centrifugation and storage at 4 °C and 40 °C. **k** Viscosity curve of the RDPM hydrogel

**Fig. 2:**
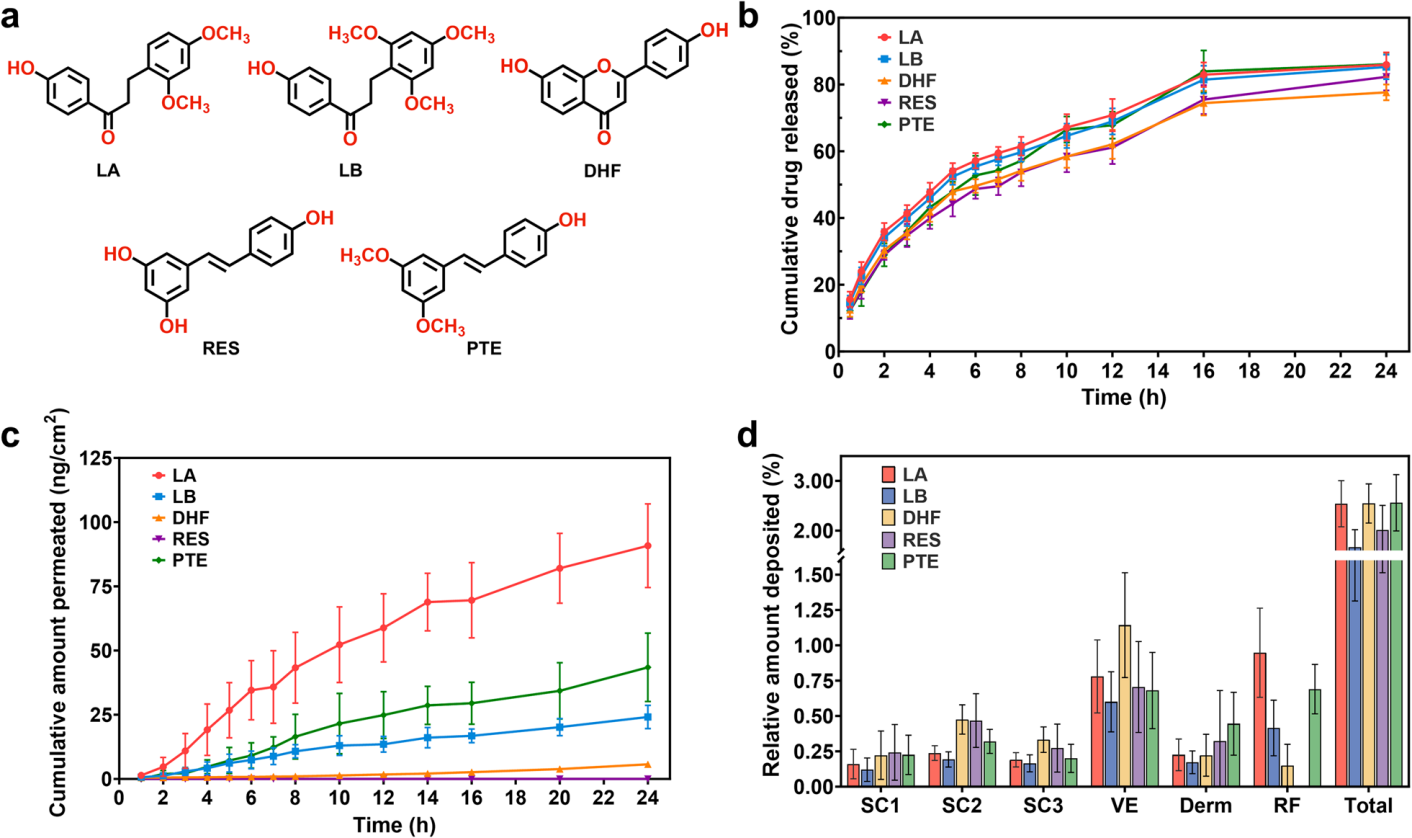
In vitro release and skin permeation of RDPM hydrogel. **a** Chemical structures of five representative bioactive constituents of *RD*: LA, LB, DHF, RES, PTE. **b** Cumulative in vitro release profiles of these constituents from the RDPM hydrogel (n = 6). **c** Cumulative skin permeation-time curves of the five constituents (n = 3). **d** Distribution and deposition levels of these compounds across different skin layers 24 h after RDPM hydrogel application. SC1: 1st tape strip; SC2: 2nd–5th tape strips; SC3: 6th–10th tape strips; VE: viable epidermis; Derm: dermis; RF: receptor fluid; Total: sum of all layers

The RDPM hydrogel was prepared through a two-step process involving the formation of polymeric micelles followed by their incorporation into a hydrogel matrix, as illustrated in Fig. 1a. The detailed formulation composition is shown in Fig. 1b. The *RD* content in the RDPM hydrogel was 1% (w/w), which falls within a commonly used range for topical semisolid formulations containing standardized botanical extracts or defined phytochemical fractions. RDPMs were prepared using the thin-film hydration method and exhibited a narrow particle size distribution (Fig. 1c), with an average hydrodynamic diameter of 17.91 ± 0.05 nm, a polydispersity index (PDI) of 0.154, and a zeta potential of −7.58 ± 1.91 mV. TEM further confirmed that the RDPMs were well-dispersed, spherical nanoparticles consistent with the results by dynamic light scattering (DLS), showing no evidence of aggregation (Fig. 1d). The encapsulation efficiencies of five representative bioactive constituents from *RD*—including three flavonoids (LA, LB, and DHF) and two stilbenoids (PTE and RES)—was generally higher than 90%, except LB (80.60%) (Fig. 1e). The chemical structures of these compounds are presented in Fig. 2a. The drug loading contents of these constituents in the RDPMs ranged from 0.038% to 0.116% (Fig. 1f).

The RDPM hydrogel was subsequently obtained by incorporating the RDPM dispersion into the gel matrix. The resulting formulation exhibited a uniform red-brown appearance and a smooth, viscous texture suitable for topical application (Fig. 1g). Incorporation of the micelles into the hydrogel matrix did not alter their morphology; the micelles retained their spherical shape and uniform distribution, similar to the original RDPMs (Fig. 1h). SEM revealed a porous surface morphology characteristic of the three-dimensional network structure typical of hydrogels (Fig. 1i). Stability evaluations, including centrifugation at 4,000 rpm for 30 min and storage at 4 °C and 40 °C for 24 h, showed that the RDPM hydrogel maintained a consistent appearance and structural integrity without phase separation or visible precipitation (Fig. 1j), indicating satisfactory physical stability. Rheological analysis demonstrated a progressive decrease in viscosity with increasing shear rate (0.1–100 s^−1^), confirming a shear-thinning (pseudoplastic) behavior (Fig. 1k). This property is advantageous for topical use, enabling easy spreading under shear during application while supporting retention on the skin surface once the shear stress is removed. Collectively, these findings demonstrate that the polymeric micelle hydrogel possesses favorable physicochemical characteristics, stability, and rheological properties, supporting its potential as a suitable vehicle for the topical delivery of *RD*.

### In vitro release and skin permeation behaviors of RDPM hydrogel

An in vitro release study was conducted to evaluate the drug release characteristics of the RDPM hydrogel. As shown in Fig. 2b, the cumulative release profiles of the five representative bioactive constituents were monitored over a 24-h period. The final cumulative release rates were 85.91% (LA), 85.24% (LB), 77.64% (DHF), 82.28% (RES), and 86.05% (PTE), indicating substantial release of all components during the study period. The release kinetics fitted well to the Higuchi model (Table S5), suggesting that Fickian diffusion was the predominant mechanism controlling the release process.

To further assess skin delivery performance, an in vitro skin permeation study was performed using full-thickness skin mounted on Franz diffusion cells. The receptor phase consisted of 50% (v/v) ethanol in PBS to maintain sink conditions for the poorly soluble *RD* constituents, as commonly used non-ionic solubilizers (e.g., Tween 80) did not provide sufficient solubilization in our preliminary tests. To evaluate whether this receptor medium compromised skin integrity, TER was compared before and after the permeation experiment (Table S6). Only minimal changes were observed, and TER values remained within the commonly accepted range for intact skin (≥ 20 kΩ·cm^2^) [21], suggesting a limited impact of the ethanol-containing receptor medium under our experimental conditions. As shown in Fig. 2c, the cumulative permeation profiles demonstrated that LA, LB, and PTE exhibited significantly higher permeation rates than DHF and RES. The concentration of RES in the receptor phase remained below the quantification limit, even after sample concentration, and was therefore regarded as negligible. After 24 h of application, the mean cumulative permeation amounts were 90.84 ng/cm^2^ (LA), 43.44 ng/cm^2^ (PTE), 24.13 ng/cm^2^ (LB), and 5.67 ng/cm^2^ (DHF), respectively. These variations in permeation behavior likely stem from differences in the levels of unencapsulated compounds in the hydrogel and the distinct physicochemical properties of each molecule, such as lipophilicity [22].

The distribution of the *RD* constituents across different skin layers was further analyzed (Fig. 2d). All tested compounds were detectable in each skin layer, with predominant accumulation in the deeper regions, including the viable epidermis and dermis. Notably, DHF and RES displayed more pronounced deposition within the viable epidermis compared with LA, LB, and PTE, which may be attributed to stronger hydrogen bonding interactions with epidermal biomolecules [23, 24]. Overall, the bioactive constituents of *RD* exhibited favorable skin absorption profiles. Specifically, LA, DHF, and PTE showed comparable skin absorption efficiencies (around 2.55%), which were higher than those of LB (1.67%) and RES (2.01%).

It should be noted that a direct comparison with unformulated *RD* (e.g., an aqueous suspension prone to precipitation) was not conducted, as the major RD bioactives exhibit extremely low solubility in both aqueous and lipid media, thereby limiting meaningful dissolution and diffusion-driven transport. Accordingly, the present study focused on assessing the permeation and deposition behaviors of *RD* constituents from the solubilized micellar hydrogel formulation. Taken together, the release and permeation results support the RDPM hydrogel as a solubilization-based and promising topical delivery approach for *RD*. Future studies incorporating direct comparisons with unformulated *RD* preparations would further strengthen the quantitative evidence for the delivery advantage conferred by the micellar hydrogel.

### Anti-aging effects of RDPM hydrogel in photoaged mice

**Fig. 3:**
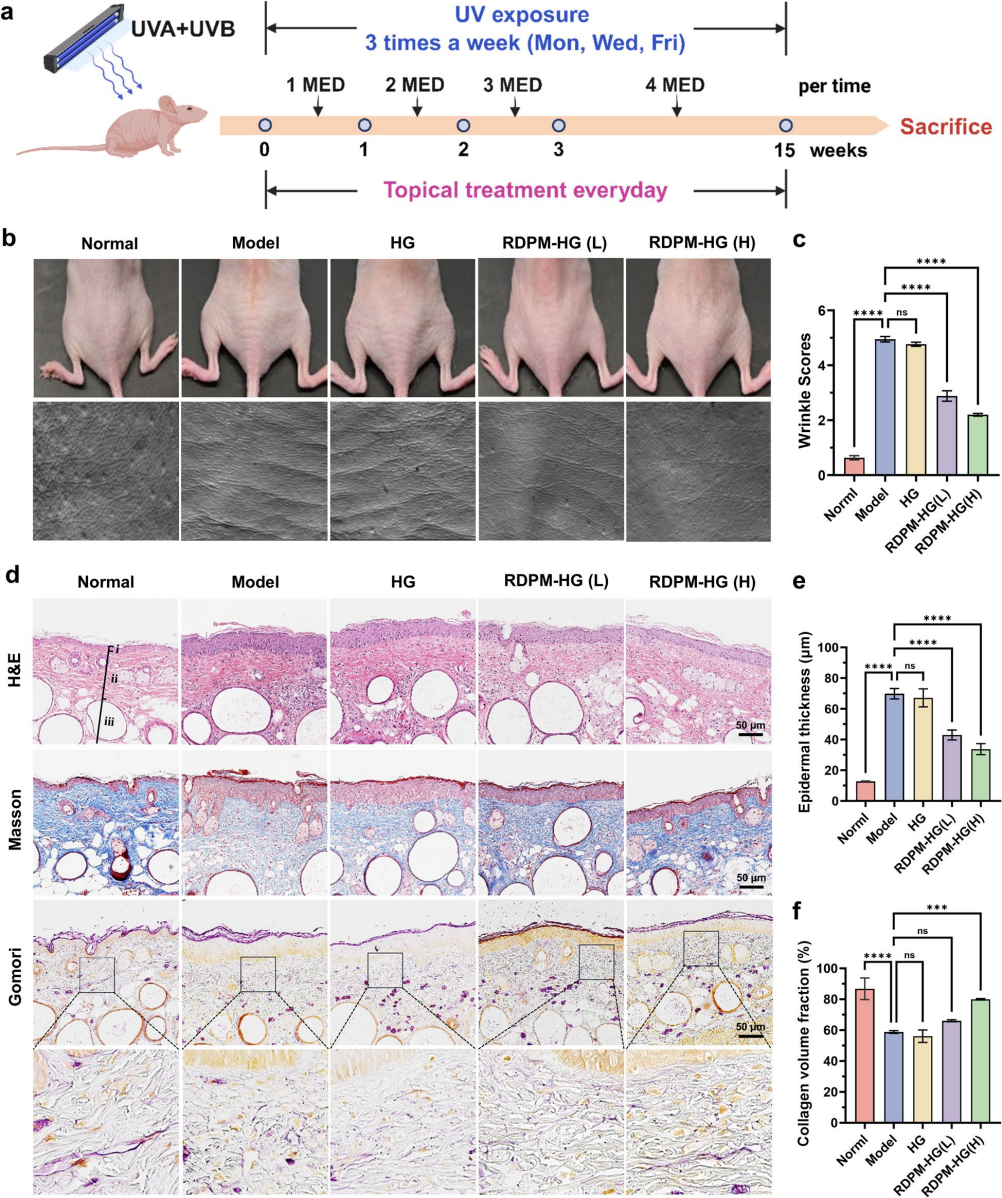
Anti-photoaging effects of RDPM hydrogel in the dorsal skin of UVB-irradiated hairless mice. **a** Schematic illustration of the experimental design, including UVB-induced photoaging model establishment and treatment protocol. **b** Representative photographs (top) and skin replicas (bottom) of the dorsal skin from UVB-irradiated hairless mice after different treatments at the end of the experimental period. Normal group: no UV exposure and no treatment; model, HG, RDPM-HG (L), and RDPM-HG (H) groups: UV exposure followed by no treatment, blank hydrogel, low-dose RDPM hydrogel, and high-dose RDPM hydrogel treatments, respectively. **c** Visual wrinkle scores of murine dorsal skin (n = 8). **d** Representative histological images of dorsal skin sections stained with H&E, Masson’s trichrome, and Gomori’s aldehyde fuchsin. Labels: (i) epidermis; (ii) dermis; (iii) subcutaneous tissue. **e** Average epidermal thickness of dorsal skin (n = 3). **f** Average collagen density of dorsal skin (n = 3). The histological staining and quantitative analyses in (**d**, **e**, **f**) were performed on randomly selected mice (n = 3 per group) prior to tissue processing. Data are presented as mean ± SD. Statistical analyses were performed using one-way ANOVA. Significant differences between groups are indicated as: ^***^p < 0.001, ^****^p < 0.0001; ns, not significant (p > 0.05)

In this study, UV irradiation was employed to induce photoaging in hairless mice (Fig. 3a), with wrinkle formation serving as a primary indicator of photoaging. After 15 weeks of UV exposure, the model group developed pronounced, deep, and elongated wrinkles on the dorsal skin compared with the normal group (Fig. 3b). Treatment with both low- and high-dose RDPM gel markedly alleviated wrinkle formation, reducing wrinkle length, depth, and number. Remarkably, the high-dose RDPM treatment nearly abolished visible wrinkles, restoring a smoother skin surface. In contrast, the blank micelle hydrogel exerted no appreciable anti-wrinkle effect. Quantitative evaluation of dorsal skin appearance, performed according to the established scoring criteria (Table S7), is presented in Fig. 3c.

Photoaged skin typically exhibits profound structural alterations, including epidermal hyperplasia, flattening of the dermal–epidermal junction (DEJ), degradation of dermal collagen, and accumulation of abnormal elastotic fibers—all of which collectively contribute to wrinkle formation [25]. Histopathological analysis with H&E staining revealed a markedly thickened epidermis and a flattened DEJ in the model group relative to the normal control, indicating disrupted epidermal-dermal contact (Fig. 3d, e). UV-induced alterations in collagen and elastic fibers were further assessed through Masson’s trichrome and Gomori’s aldehyde fuchsin staining. In normal skin, collagen fibers appeared dense, parallel, and well-organized, whereas in the model group they were fragmented, irregularly aligned, and reduced in density (Fig. 3d, 3f). Moreover, the elastic fiber network, which was finely interwoven with collagen in normal skin, exhibited severe damage after UV exposure, characterized by clumped, thickened, and tangled fibers—a hallmark of solar elastosis (Fig. 3d). Topical application of RDPM hydrogel effectively mitigated these photoaging-related structural alterations in a dose-dependent manner. In particular, the high-dose RDPM group displayed a thinner epidermis, a wavy DEJ with well-defined dermal papillae, and an intact dermis with organized collagen and elastic fibers, closely resembling the morphology of normal skin. Therefore, RDPM hydrogel exerted a pronounced protective effect against UV-induced photoaging.

### Metabolomic profiling and pathway analysis

**Fig. 4:**
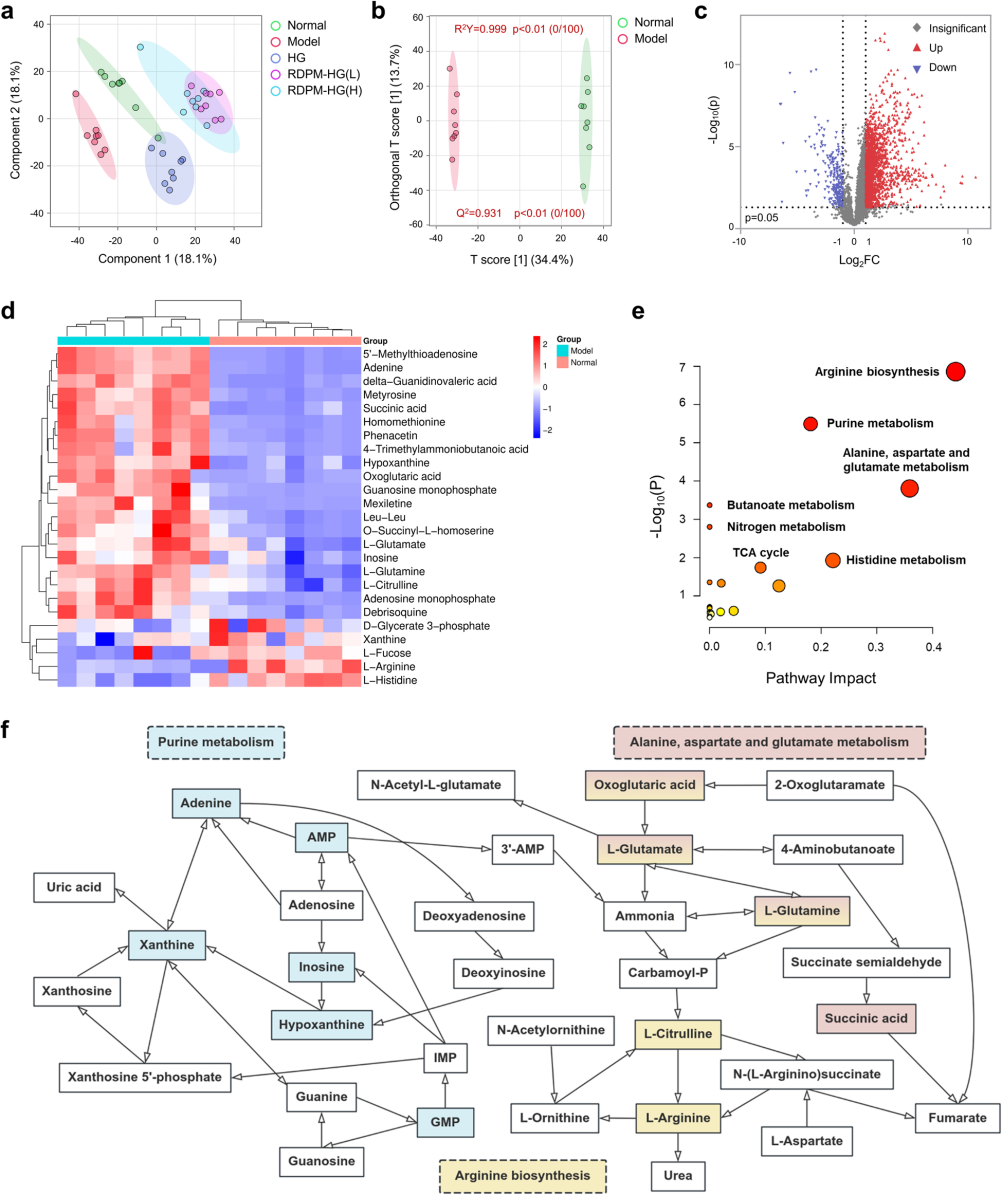
Metabolomics analysis of dorsal skin tissues in UV-irradiated hairless mice with different treatments. **a** PLS-DA score plot showing clear separation of metabolic profiles among the normal, model, HG, RDPM-HG (L), and RDPM-HG (H) groups. Normal group: unirradiated and untreated; model, HG, RDPM-HG (L), and RDPM-HG (H) groups: UVB-irradiated and treated with no formulation, blank hydrogel, low-dose RDPM hydrogel, or high-dose RDPM hydrogel, respectively. **b** OPLS-DA score plot comparing the normal and model groups. **c** Volcano plot illustrating differentially expressed metabolites between the normal and model groups. **d** Hierarchical clustering heatmap of the top 25 differentially expressed metabolites identified between the normal and model groups, whose alterations were normalized upon RDPM hydrogel treatment. **e** Metabolomic pathway enrichment analysis of the 25 significantly altered metabolites. **f** Topology analysis of the key metabolic pathways significantly modulated by RDPM hydrogel treatment

To elucidate the biochemical mechanisms underlying the protective effects of the RDPM hydrogel against UV-induced photoaging, an untargeted LC–MS/MS-based metabolomic analysis was performed. An initial PCA was first performed to obtain an overview of global metabolic variations (Fig. S2). The clear separation between the normal and model groups suggested pronounced UV-induced metabolic alterations. A subsequent PLS-DA was employed to better resolve intergroup metabolic differences (Fig. 4a). The PLS-DA score plot revealed well-separated clusters among the experimental groups, verifying pronounced metabolic alterations associated with photoaging. Importantly, the RDPM hydrogel-treated groups, particularly the high-dose formulation, exhibited a trend toward normalization, indicating amelioration of UV-induced metabolic disruptions.

To further discriminate between the normal and model groups and identify key differential metabolites, an OPLS-DA model was constructed (Fig. 4b). Model reliability was evaluated using the R^2^Y and Q^2^ parameters, where R^2^Y and Q^2^ values greater than 0.5 and a difference less than 0.3 indicate good model stability and predictive capability. In this study, the OPLS-DA model yielded R^2^Y = 0.999 and Q^2^ = 0.931, confirming its robustness and strong predictive performance. Differential metabolites were visualized in a volcano plot, and statistical significance was defined by VIP > 1.0 and p < 0.05 (Fig. 4c). A total of 59 metabolites showed significant differences between the normal and model groups, among which 25 metabolites were regulated toward normal levels following RDPM hydrogel treatment (Fig. 4d and S3, Table S8). Pathway enrichment analysis of these metabolites revealed significant alterations in arginine biosynthesis, purine metabolism, alanine/aspartate/glutamate metabolism, and histidine metabolism, with the first three pathways showing the most pronounced responses to RDPM intervention (Fig. 4e).

The alterations in the three key metabolic pathways are summarized in Fig. 4f and S2. Within the purine metabolism pathway, photoaged skin displayed elevated adenine, AMP, inosine, hypoxanthine, and GMP, accompanied by a reduction in xanthine, suggesting disrupted nucleotide turnover. In the arginine biosynthesis pathway, UV exposure led to a marked depletion of arginine alongside accumulation of its precursors (oxoglutaric acid, glutamate, glutamine, and citrulline), indicative of an imbalance in nitrogen cycle. In alanine, aspartate, and glutamate metabolism, elevated levels of oxoglutaric acid, glutamate, glutamine, and succinic acid were observed, indicative of tricarboxylic acid (TCA) cycle disturbance and mitochondrial stress in photoaged tissues. Topical RDPM hydrogel treatment was associated with a clear normalization of these changes, lowering elevated purine intermediates and TCA-related metabolites while restoring depleted metabolites such as arginine and xanthine toward baseline levels. Collectively, these results indicate that RDPM hydrogel alleviates photoaging-associated metabolic perturbations linked to nucleotide turnover, nitrogen balance and mitochondrial energy metabolism, providing mechanistic support for its protective effects against UV-induced skin photoaging.

### Skin irritation evaluation of RDPM hydrogel

**Fig. 5:**
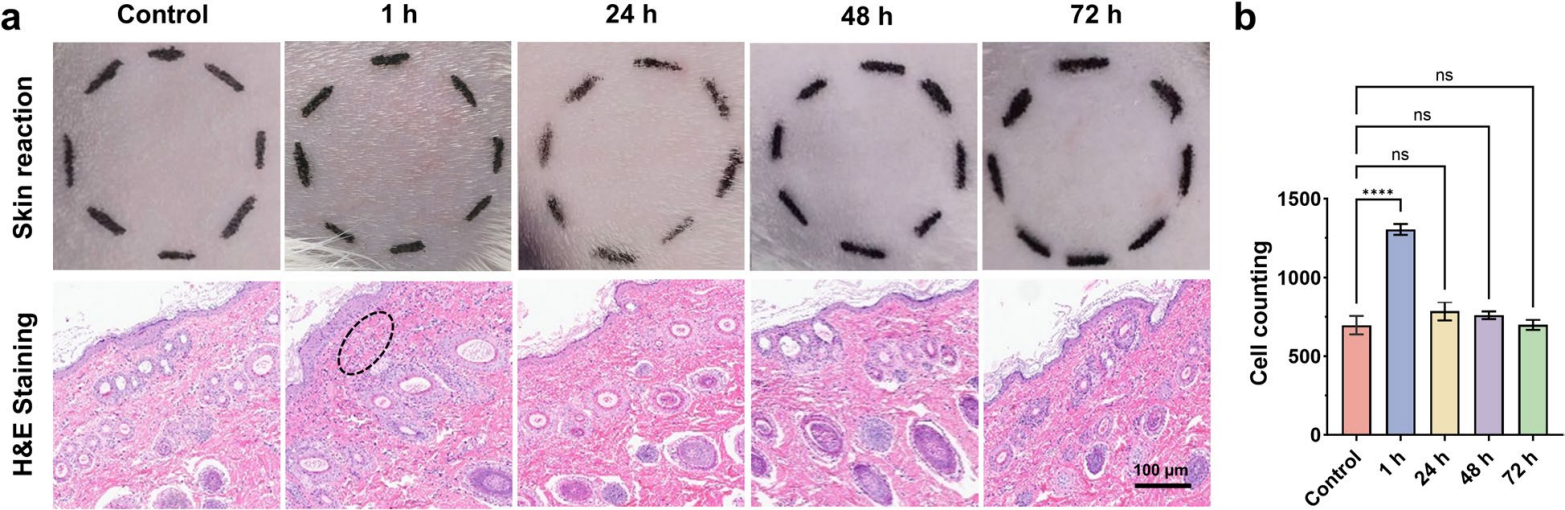
Evaluation of skin irritation in guinea pigs following topical application of the RDPM hydrogel. **a** Top: Macroscopic assessment of erythema and edema on guinea pig skin over 72 h after topical application of the RDPM hydrogel. Bottom: Representative histopathological images of skin tissues collected from the application sites at designated time points. **b** Quantitative analysis of dermal cell density within a defined region of interest (450 μm × 150 μm, confined to the dermal layer) based on histological sections (n = 3). Data are presented as mean ± SD (n = 3). Statistical significance was determined using one-way ANOVA. ^****^p < 0.0001; ns, not significant (p > 0.05)

A skin irritation test was performed in guinea pigs to evaluate the dermal safety of the RDPM hydrogel. Animals were monitored for erythema and edema for 72 h following topical application (Fig. 5a). Within one hour of administration, two of the four guinea pigs exhibited mild erythema, which resolved completely within 24 h and did not recur throughout the observation period. The remaining two animals showed no visible signs of erythema or edema during the 72-h evaluation. The primary irritation index (PII) was calculated to be 0.125, which falls within the non-irritating range (0.0–0.4), indicating that the RDPM hydrogel caused no evident irritation to the skin.

Histopathological analysis was conducted on skin tissues collected from treated sites at each time point (Fig. 5a). Microscopic examination revealed no noticeable structural alterations in the epidermis. However, transient inflammatory cell infiltration was observed in the upper dermal layer 1 h post-application, which resolved by 24 h. Quantitative analysis of dermal cellular density within a defined region of interest (450 μm × 150 μm) using ImageJ software showed a peak in cell number at 1 h, followed by normalization to baseline levels after 24 h (Fig. 5b). These histological observations were consistent with the macroscopic skin responses, collectively demonstrating that the RDPM hydrogel elicited only a minimal, transient, and self-resolving skin reaction, confirming its good local biocompatibility for topical use.

## Discussion

*RD* possesses well-documented medicinal properties, including promoting blood circulation, relieving pain, resolving blood stasis, arresting bleeding, and enhancing tissue regeneration and wound healing. As a domestically sourced alternative to *Sanguis Draconis* (Xue Jie), which is listed in the Chinese Pharmacopoeia, *RD* exhibits comparable therapeutic efficacy but differs in botanical origin and possesses distinct chemical compositions and pharmacological properties [26]. Among its diverse bioactivities, the potent antioxidant, anti-inflammatory, and tissue-repairing properties of *RD* are particularly relevant, as directly counteract the fundamental pathological processes underlying skin photoaging, such as oxidative stress, inflammation, and extracellular matrix degradation. Based on these mechanistic correlations, we hypothesized that *RD* could exert protective effects against UV-induced skin photoaging. To preliminarily test this hypothesis, we investigated the anti-senescence effects of *RD* on UV-irradiated human dermal fibroblasts (Fig. S4). The results showed that *RD* markedly reduced the proportion of senescent cells—identified by senescence-associated β-galactosidase (SA-β-gal) staining—in a dose-dependent manner following UV exposure. Moreover, *RD* treatment significantly restored fibroblast proliferation, as evidenced by increased cell density after irradiation. This indicates that *RD* can attenuate UV-induced cellular senescence, thereby offering protective and rejuvenating potential against skin photoaging.

The major bioactive constituents of *RD*, primarily flavonoids and stilbenoids, exhibit intrinsically poor solubility in both aqueous and lipid media, which poses a significant challenge for formulation development and limits their bioavailability. In this study, an RDPM hydrogel was developed to enhance solubility and facilitate skin delivery. The poloxamer-based micellar system achieved high encapsulation efficiencies exceeding 90% for most constituents (except LB, 80.60%), facilitating effective solubilization of *RD*. Incorporation of the micelles into a carbomer-based hydrogel matrix produced a formulation with desirable spreadability and texture, while preserving micellar integrity and stability. The in vitro release study demonstrated that more than 80% of the encapsulated constituents (except DHF, 77.64%) were released within 24 h, and the release data exhibited a strong correlation with the Higuchi model. This finding indicates that the release mechanism was predominantly governed by Fickian diffusion, wherein bioactive molecules gradually migrated from the micellar domains through the hydrogel matrix into the external medium. The dominance of diffusion-controlled release, rather than hydrogel erosion or micelle disintegration, implies that the hydrogel network provided a stable and continuous diffusion pathway while maintaining micellar integrity, thereby ensuring sustained and predictable release kinetics. Such a release profile is particularly advantageous for topical formulations, as it supports consistent drug availability at the application site and may enhance therapeutic efficacy while minimizing systemic exposure.

In vitro skin permeation studies of RDPM hydrogel revealed that the main active flavonoid and stilbenoid constituents achieved approximately 2% skin absorption efficiency, a level generally considered typical for topical delivery formulations. Notably, most absorbed molecules were localized in deeper skin layers, including the viable epidermis and dermis, indicating effective penetration through the SC. Notably, RES and HDF displayed pronounced deposition in the viable epidermis, plausibly due to hydrogen bond-mediated interactions between their polyphenolic hydroxyl groups and epidermal proteins or phospholipids [23, 24]. Such localized deposition is advantageous for maintaining therapeutic levels at target skin layers implicated in photoaging.

Photoaged skin is characterized by wrinkle formation and histological changes, including epidermal hyperplasia, flattening of the DEJ, collagen degradation, and elastotic degeneration [25]. Topical administration of RDPM hydrogel markedly ameliorated these photoaging-associated morphological and structural alterations in UV-exposed mice, whereas the blank hydrogel exerted no appreciable effect. These results collectively confirm that the active constituents of *RD* delivered via RDPM hydrogel effectively reversed UV-induced photoaging, supporting our hypothesis regarding its protective potential.

To further elucidate the biochemical mechanisms associated with the observed protection, untargeted metabolomic profiling was conducted. Compared with non-irradiated skin, photoaged skin displayed marked perturbations in purine and amino acid metabolism, signifying UV-induced metabolic reprogramming. In the purine metabolism pathway, increased adenine, AMP, inosine, hypoxanthine, and GMP levels are consistent with accelerated nucleotide turnover and a higher demand for DNA/RNA maintenance under UV stress. The concomitant decline in xanthine may suggest impaired xanthine dehydrogenase/oxidase activity, potentially disturbing reactive oxygen species (ROS) generation and redox balance [27, 28]. Notably, RDPM hydrogel treatment was associated with normalization of these purine metabolites, implying a reduced nucleotide-damage burden and a shift toward re-balanced purine catabolism. Given that *RD* contains abundant antioxidant constituents such as flavonoids and stilbenoids, these changes are plausibly explained by attenuation of UV-induced oxidative stress (e.g., via ROS scavenging and suppression of pro-oxidative signaling), thereby facilitating recovery of nucleotide metabolic homeostasis.

Alterations in the arginine biosynthesis pathway were also prominent, underscoring the metabolic relevance of arginine to cutaneous inflammation and tissue repair. The depletion of arginine in photoaged skin may reflect increased utilization for nitric oxide (NO) production, a mediator implicated in oxidative and inflammatory responses [29]. Meanwhile, the accumulation of intermediates such as citrulline and glutamine suggests a pathway-level imbalance, compatible with impaired arginine recycling [30]. Such dysregulation could compromise collagen synthesis and repair capacity, both of which are arginine-dependent [31, 32]. RDPM hydrogel treatment was accompanied by restoration of arginine levels and a trend toward normalization of related intermediates, supporting a role in re-equilibrating arginine biosynthesis and arginine-NO homeostasis. This interpretation is consistent with improved tissue-repair microenvironments, in which more balanced arginine-NO signaling favors collagen synthesis, angiogenesis, and regenerative processes.

Furthermore, elevated oxoglutaric acid, glutamate, and succinic acid mapped to alanine/aspartate/glutamate metabolism and the TCA cycle are indicative of mitochondrial stress and perturbed energy metabolism in photoaged skin [33]. Mitochondrial dysfunction is a recognized contributor to premature senescence under chronic UV exposure [34]. Succinic acid, in addition, is increasingly appreciated as a signaling metabolite capable of stabilizing HIF-1α and promoting pro-inflammatory programs, which may aggravate the chronic inflammatory milieu of photoaging [35]. RDPM hydrogel treatment was associated with normalization of these metabolites, suggesting improved mitochondrial metabolic balance and attenuation of pseudohypoxic/inflammatory signaling. Mechanistically, these metabolomic patterns are compatible with reduced oxidative damage to mitochondrial enzymes and dampened inflammatory cascades following *RD* intervention. Taken together, the metabolomic data provide systems-level biochemical evidence that *RD* intervention is closely associated with reversal of UV-disrupted metabolic pathways. Rather than establishing a single causal direction, these pathway normalizations should be viewed as mechanistically informative readouts consistent with the known antioxidative, anti-inflammatory, and tissue-repairing activities of *RD*. Overall, RDPM hydrogel treatment correlated with improvement of redox balance, re-equilibration of arginine-NO-related metabolism, and preservation of mitochondrial metabolic integrity, alongside restoration of purine and amino-acid fluxes in photoaged skin.

The skin irritation assessment of the RDPM hydrogel demonstrated good local biocompatibility, confirming its suitability for topical application. The transient, self-resolving and very slight erythema observed after RDPM hydrogel application suggests a short-term adaptive inflammatory response. Such immediate but reversible dermal reactions are commonly associated with initial immune surveillance and local vascular responses triggered by mild physicochemical stimulation at the skin surface [36]. The rapid disappearance of inflammatory infiltrates and the absence of structural alterations in the epidermis further confirm that RDPM hydrogel induces only minimal, non-pathological stimulation, consistent with its classification as non-irritating (PII = 0.125). From a pharmaceutical standpoint, this transient response may even be beneficial, as mild local inflammation can enhance microcirculation and promote transdermal absorption of active compounds without compromising barrier integrity. Therefore, these findings substantiate the dermal safety profile of the RDPM hydrogel and provide a preclinical foundation for its further development as a topical therapeutic.

## Conclusion

This study successfully developed a polymeric micelle hydrogel (i.e., RDPM hydrogel) that overcomes the intrinsic solubility and bioavailability limitations of *RD*’s major bioactive constituents, enabling efficient dermal absorption and potent protection against UV-induced photoaging. Untargeted metabolomic profiling revealed that RDPM treatment was associated with a marked normalization of UV-perturbed metabolic networks (especially purine, arginine, and alanine/aspartate/glutamate pathways), consistent with improved redox balance, restored arginine-NO signaling, and preservation of mitochondrial function. These findings provide mechanistic validation of *RD*’s pharmacological efficacy and establish a modern scientific rationale for its use in skin protection and repair. From a broader perspective, RDPM hydrogel represents a paradigm for precision engineering of botanical formulations that bridges traditional medicine and modern pharmaceutics, supporting their translation into clinically relevant, mechanism-driven therapeutics.

## Supplementary Information

Supplementary Material 1.

## Data Availability

Data will be made available on request.

## Abbreviations

UV: Ultraviolet
ROS: Reactive oxygen species
MAPK: Mitogen-activated protein kinase
NF-κB: Nuclear factor kappa B
MMP: Matrix metalloproteinase
ECM: Extracellular matrix
TGF-β: Transforming growth factor-β
RD: *Resina* *Draconis*
Nrf2: Nuclear factor erythroid 2-related factor 2
HO-1: Heme oxygenase-1
SOD: Superoxide dismutase
GSH-Px: Glutathione peroxidase
AP-1: Activator protein-1
TNF-α: Tumor necrosis factor-α
IL-1β: Interleukin-1β
RDPM: RD-loaded polymeric micelle
LA: Loureirin A
LB: Loureirin B
DHF: 7,4′-dihydroxyflavone
PTE: Pterostilbene
RES: Resveratrol
SC: Stratum corneum
MED: Minimal erythema dose
H&E: Hematoxylin–eosin
HMDB: Human Metabolome Database
KEGG: Kyoto Encyclopedia of Genes and Genomes
DEJ: Dermal–epidermal junction
SA-β-Gal: Senescence-associated β-galactosidase
TCA: Tricarboxylic acid
PII: Primary irritation index
NO: Nitric oxide
