# Supplementary material for: Polymeric micelle hydrogel of Resina Draconis enables mechanistically targeted protection against skin photoaging
Source: Chin Med. 2026 Feb 16;21:70. doi: 10.1186/s13020-026-01344-w (PMC12911310; doi:10.1186/s13020-026-01344-w)
Supplement: Supplementary file 1 — Supplementary Material 1. [file 13020_2026_1344_MOESM1_ESM.pdf]

Supporting Information

Polymeric micelle hydrogel of *Resina Draconis* enables mechanistically targeted protection against skin photoaging

Mengzhu Liu[a,b,1], Yuqing Huang[b,c,1], Wanyang Sun[d], Yuqi Wang[b,e], Xiu Yu[f], Huichao Xie[b], Pingtian Ding[b], Yuanyuan Xie[c,*], Keda Zhang[b,*]

[a] School of Pharmacy, Shenzhen University Medical School, Shenzhen University, Shenzhen, 518055, China.

[b] College of Pharmacy, Shenzhen Technology University, Shenzhen 518118, China.

[c] School of Chinese Materia Medica, Guangdong Pharmaceutical University, Guangzhou 510006, China

[d] State Key Laboratory of Bioactive Molecules and Druggability Assessment/Guangdong Engineering Research Center of Traditional Chinese Medicine & Disease Susceptibility/Guangdong Engineering Research Center of Traditional Chinese Medicine & Health Products/Institute of Traditional Chinese Medicine and Natural Products, College of Pharmacy, Jinan University, Guangzhou 510632, China

[e] School of Pharmacy, Shenyang Pharmaceutical University, Shenyang 110016, China.

[f] Department of Pulmonary and Critical Care Medicine, Shenzhen Key Laboratory of Respiratory Diseases, Shenzhen Clinical Research Center for Respiratory Disease, Shenzhen Institute of Respiratory Diseases, Shenzhen People's Hospital (The First Affiliated Hospital, Southern University of Science and Technology, The Second Clinical Medical College, Jinan University), Shenzhen, 518020, China.

[*] Corresponding authors.

E-mail addresses: zhangkeda@sztu.edu.cn (K. Zhang), yuanyuan8078@163.com (Y. Xie)

[1] The authors contributed equally to this work.

Cellular Senescence Assay

Human dermal fibroblasts (HDFs; 5 × 10^4 cells per well) were seeded into six-well plates and cultured for 24 h in Dulbecco's Modified Eagle Medium (DMEM) supplemented with 10% fetal bovine serum (FBS) and 1% penicillin–streptomycin. Cells were maintained at 37 °C in a humidified incubator with 5% CO_2. To induce quiescence, the medium was replaced with serum-free DMEM for 12 h prior to treatment. Following serum starvation, the cells were gently washed with PBS and exposed to UV radiation using a UVB lamp (UVB-313EL, Q-Lab, USA) at a dose of 70 mJ/cm^2. The irradiation dose was monitored using a digital UV meter (SDR2040, Speedre, Shenzhen, China) with a detection range of 280–400 nm. Subsequently, the cells were cultured with *RD* at different concentrations of 0, 5, or 15 μg/mL in serum-free DMEM for 48 h. Control cells were maintained in serum-free DMEM without UV irradiation. Following treatment, cells were fixed and stained using a senescence-associated β-galactosidase (SA-β-gal) staining kit (Cell Signaling Technology, USA) according to the manufacturer's instructions. Images were acquired using an inverted microscope (Leica DMI1, Leica Microsystems, Wetzlar, Germany), and senescent cells were quantified based on the presence of characteristic blue staining.

Sequential re-extraction for extraction-efficiency validation

To verify extraction completeness for skin-deposition quantification, a sequential re-extraction procedure was performed for the stratum corneum (SC), viable epidermis, and dermis samples prepared as described in Section 2.7 (SC collected by 10 tape strips; viable epidermis obtained by careful scraping; dermis finely minced). After collecting the first extract (E1), the remaining SC tapes or tissue residues (viable epidermis/dermis) were subjected to two additional extraction cycles under the same conditions to obtain E2 and E3. To minimize carryover of residual extract from the previous cycle, a water-rinse step was included between successive extractions. All extract fractions were analyzed by LC-MS/MS. For each analyte in each skin layer, the amount recovered in each fraction (E1, E2, and E3) was calculated from the measured concentration and the corresponding extraction volume. Extraction completeness was assessed as the percentage recovered in E1 relative to the total amount recovered across E1–E3 (mass-balance approach).

Table S1. Quantitative analysis of five representative bioactive constituents in *RD*.

Constituents	Content (mg/g)
LA	13.82
LB	9.62
DHF	5.59
RES	4.45
PTE	9.10

Table S2. Sequential re-extraction recovery of *RD* constituents from skin layers (mean ± SD, %, n = 3)[a].

Skin layer	Extraction	LA	LB	DHF	RES	PTE
SC	1st	99.19 ± 0.24	99.45 ± 0.32	98.90 ± 0.95	100.00	100.00
	2nd	0.81 ± 0.24	0.55 ± 0.32	1.10 ± 0.95	n.q.	n.q.
	3rd	n.q.	n.q.	n.q.	n.q.	n.q.
Viable epidermis	1st	99.23 ± 1.05	99.35 ± 0.19	98.36 ± 0.25	100.00	100.00
	2nd	0.77 ± 1.05	0.65 ± 0.19	1.64 ± 0.25	n.q.	n.q.
	3rd	n.q.	n.q.	n.q.	n.q.	n.q.
Dermis	1st	97.50 ± 0.95	100.00	100.00	100.00	100.00
	2nd	2.50 ± 0.95	n.q.	n.q.	n.q.	n.q.
	3rd	n.q.	n.q.	n.q.	n.q.	n.q.

[a] Values are expressed as the percentage relative to the total amount recovered across the 1st–3rd extractions within each skin layer. n.q., not quantifiable (below the limit of quantification).

Table S3. Erythema and edema draize scales for scoring skin irritation.

Value	Erythema and eschar formation	Value	Edema formation
0	No erythema	0	No edema
1	Very slight erythema (barely perceptible), edges of area not well defined	1	Very slight edema (barely perceptible), edges of area not well defined)
2	Slight erytherma (pale red in color and deges definable)	2	Slight edema (edges of area well defined by definite raising)
3	Moderate-to-severe erythema (defined in color and area well defined)	3	Moderate edema (raised ~1 mm)
4	Severe erythema (beet to crimson red) to slight eschar formation (injuries in depth)	4	Severe edema (raised more than 1 mm and extending beyond area of exposure)

Table S4. Descriptive rating for primary skin irritation index (PII)

PII value[a]	Classification
<0.5	Non-irritating
From 0.5 to <2.0	Slightly irritating
From 2.0 to <5.0	Moderately irritating
From 5.0 to 8.0	Severely irritating

[a] PII value was calculated by adding the average erythema and edema scores for the 1 h, 24 h, 48 h, and 72 h scoring intervals and dividing by the number of evaluation intervals.

Table S5. Higuchi model fitting parameters for the *in vitro* release of five representative bio-active constituents from the RDPM hydrogel.

Constituents	Higuchi model	
	**Fitting equation**[a]	**R^2**
LA	$Q_n = 17.25t^{1/2} + 10.95$	0.9566
LB	$Q_n = 17.26t^{1/2} + 9.32$	0.9627
DHF	$Q_n = 15.89t^{1/2} + 7.62$	0.9619
RES	$Q_n = 16.96t^{1/2} + 4.31$	0.9833
PTE	$Q_n = 18.86t^{1/2} + 3.14$	0.9691

[a] Q_n is the cumulative drug release fraction (expressed in %) at time t.

Table S6. Transdermal electrical resistance (TER) of the skin before and after *in vitro* skin permeation study.

No.	TER (kΩ·cm²)	
	**Pre-study**	**Post-study**
1	26.7	25.3
2	30.3	31.3
3	34.5	27.4

Table S7. Grading scale for evaluation of photoaging.

Grade	Evaluation criteria
0	No wrinkles or laxity; fine striations running the length of the body
1	Fine striations
2	Disappearance of all fine striations
3	Shallow wrinkles
4	A few deep wrinkles and laxity
5	Increased deep wrinkles
6	Severe wrinkles; development of tumors/lesions

Table S8. LC–MS/MS characteristics and identification of key differential metabolites between the Normal and Model groups.

NO.	RT [min]	m/z	Formula	Δ(ppm)	Proposed identity	Adduct type	HMDB ID	KEGG ID
1	0.75	145.01365	$C_5H_6O_5$	-2.37	Oxoglutaric acid	$[M-H]^-$	HMDB0000208	C00026
2	1.21	117.01841	$C_4H_6O_4$	2.37	Succinic acid	$[M-H]^-$	HMDB0000254	C00042
3	0.67	152.88315	$C_6H_9N_3O_2$	-1.18	L-Histidine	$[M-H]^-$	HMDB0000177	C00135
4	0.99	346.05679	$C_{10}H_{14}N_5O_7P$	-2.13	Adenosine monophosphate	$[M-H]^-$	HMDB0000045	C00020
5	0.82	153.04113	$C_5H_4N_4O_2$	2.83	Xanthine	$[M+H]^+$	HMDB0000292	C00385
6	1.00	364.06614	$C_{10}H_{14}N_5O_8P$	2.83	Guanosine monophosphate	$[M+H]^+$	HMDB0001397	C00144
7	0.63	196.87859	$C_6H_{14}N_4O_2$	-0.22	L-Arginine	$[M+Na]^+$	HMDB0000517	C00062
8	3.08	136.06209	$C_5H_5N_5$	2.37	Adenine	$[M+H]^+$	HMDB0000034	C00147
9	1.27	137.04606	$C_5H_4N_4O$	1.97	Hypoxanthine	$[M+H]^+$	HMDB0000157	C00262
10	3.86	245.18679	$C_{12}H_{24}N_2O_3$	3.33	Leu-Leu	$[M+H]^+$	HMDB0028933	C11332
11	3.08	298.09771	$C_{11}H_{15}N_5O_3S$	2.95	5'-Methylthioadenosine	$[M+H]^+$	HMDB0001173	C00170
12	9.54	180.13879	$C_{11}H_{17}NO$	2.79	Mexiletine	$[M+H]^+$	HMDB0014523	C07220
13	3.05	146.11798	$C_7H_{15}NO_2$	2.95	4-Trimethylammoniobutanoic acid	$[M+H]^+$	HMDB0001161	C01181
14	0.67	146.04527	$C_5H_9NO_4$	-3.05	L-Glutamate	$[M-H]^-$	HMDB0000148	C00025
15	0.68	147.07679	$C_5H_{10}N_2O_3$	2.65	L-Glutamine	$[M+H]^+$	HMDB0000641	C00064
16	0.67	176.10347	$C_6H_{13}N_3O_3$	2.83	L-Citrulline	$[M+H]^+$	HMDB0000904	C00327
17	0.80	184.98551	$C_3H_7O_7P$	-1.02	D-Glycerate 3-phosphate	$[M-H]^-$	HMDB0060180	C00197
18	1.40	160.10854	$C_6H_{13}N_3O_2$	3.09	delta-Guanidinovaleric acid	$[M+H]^+$	HMDB0250978	C13688
19	3.51	176.11883	$C_{10}H_{13}N_3$	3.48	Debrisoquine	$[M+H]^+$	HMDB0006543	C13650
20	1.24	218.06723	$C_8H_{13}NO_6$	0.7	O-Succinyl-L-homoserine	$[M-H]^-$	HMDB0255868	C01118
21	1.99	164.0744	$C_6H_{13}NO_2S$	2.59	Homomethionine	$[M+H]^+$	HMDB0030406	C17213
22	3.57	180.10249	$C_{10}H_{13}NO_2$	3.27	Phenacetin	$[M+H]^+$	HMDB0256387	C07591
23	2.70	196.09744	$C_{10}H_{13}NO_3$	3.19	Metyrosine	$[M+H]^+$	HMDB0014903	C07921
24	8.55	165.07619	$C_6H_{12}O_5$	2.58	L-Fucose	$[M+H]^+$	HMDB0000174	C01019
25	1.27	269.08866	$C_{10}H_{12}N_4O_5$	2.54	Inosine	$[M+H]^+$	HMDB0000195	C00294

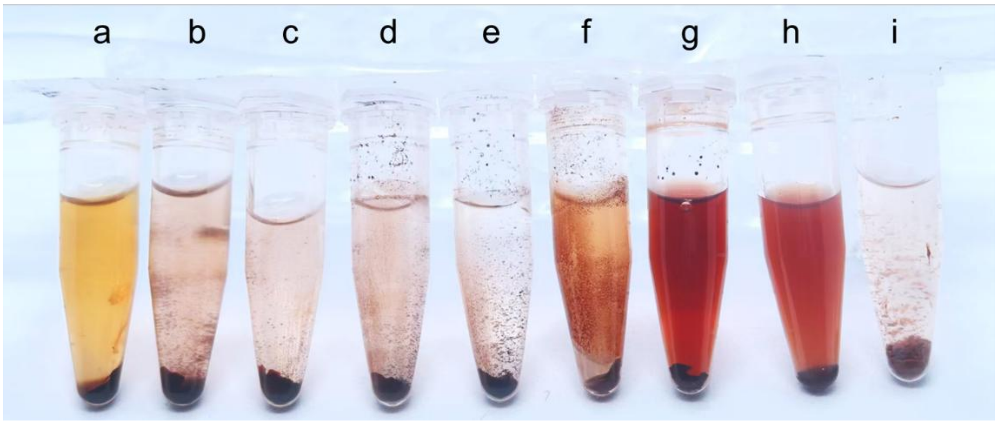

Fig. S1. Visual assessment of RD solubility (1% w/w) in various solvents and delivery systems. a) Sesame oil; b) Liquid paraffin; c) Dimethicone; d) Ultrapure water; e) 10% glycerol-water solution; f) 10% propylene glycol-water solution; g) micellar solution (10% Tween 80 in water); h) Microemulsion composed of isopropyl myristate (15%), Tween 80 (9.6%), Span 80 (6.4%), Transcutol (4%), and ultrapure water (65%); i) Liposome solution prepared with soybean lecithin (50 mM).

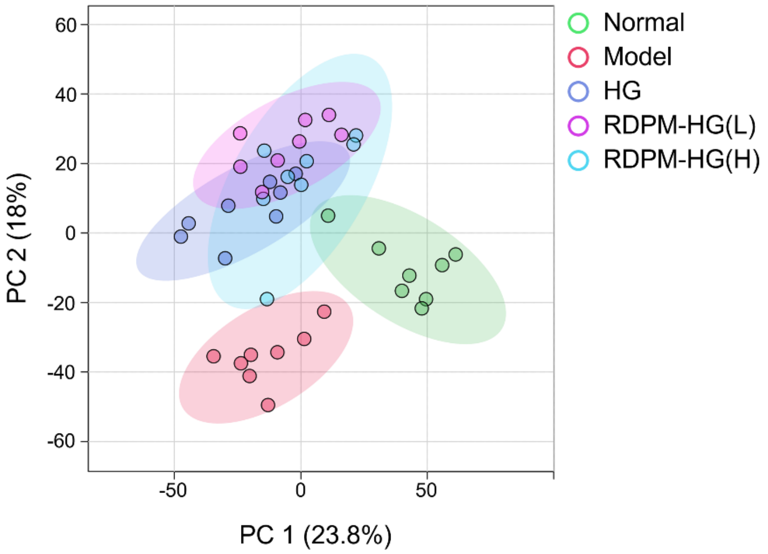

Fig. S2. PCA score plot revealing the global metabolic distribution of UV-irradiated hairless mouse skin under different treatments. Normal group: unirradiated and untreated; Model, HG, RDPM-HG (L), and RDPM-HG (H) groups: UV-irradiated and treated with no formulation, blank hydrogel, low-dose RDPM hydrogel, or high-dose RDPM hydrogel, respectively.

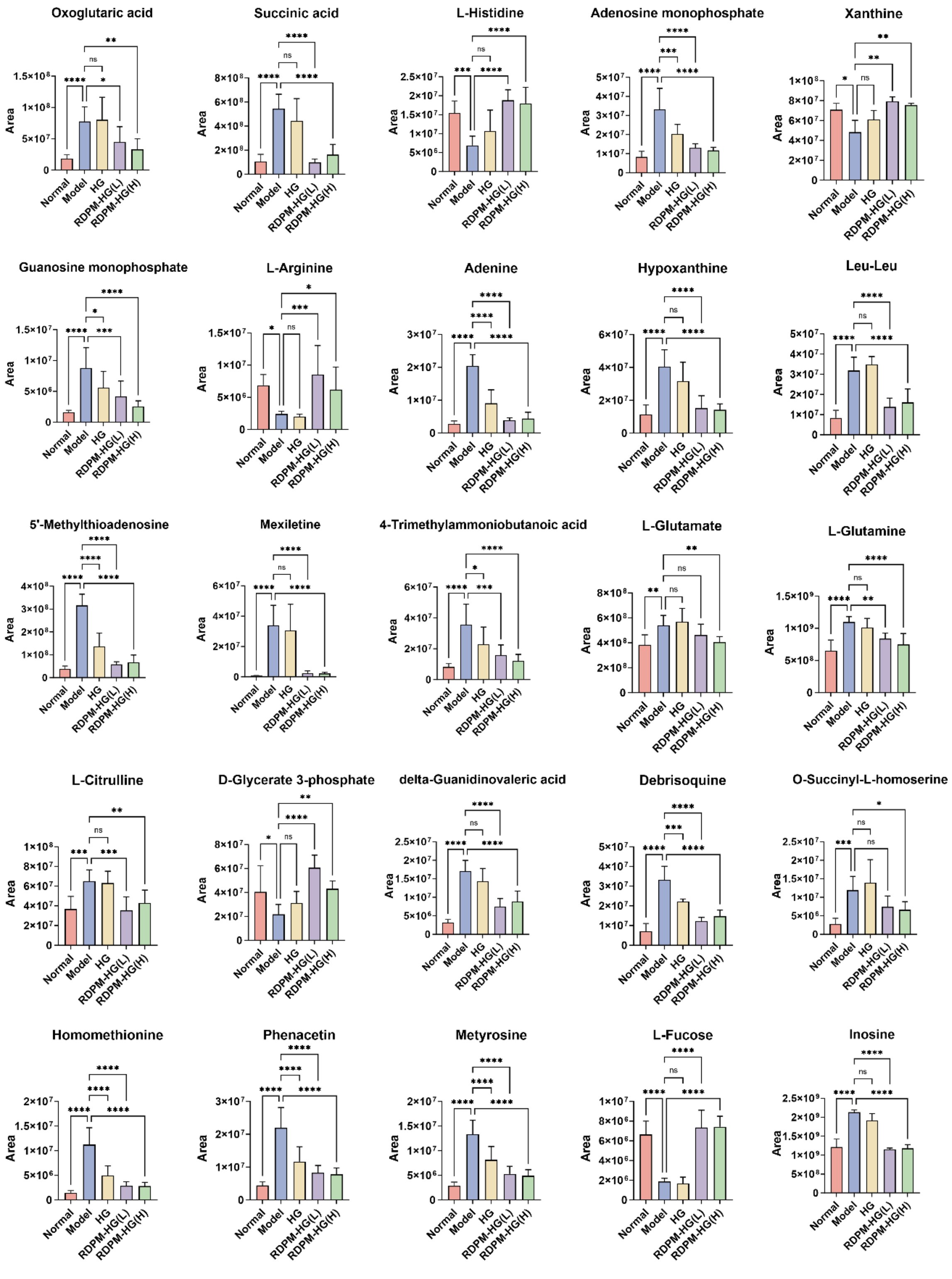

Fig. S3. Expression levels of key differential metabolites in UVB-irradiated hairless mice following different treatments. Normal group: no UV exposure and no treatment; Model, HG, RDPM-HG (L), and RDPM-HG (H) groups: UV exposure followed by no treatment, blank hydrogel, low-dose RDPM hydrogel, and high-dose RDPM hydrogel treatments, respectively. Data are presented as mean ± SD (n = 8). Statistical analysis was performed using one-way ANOVA. $^{*}p < 0.05$, $^{**}p < 0.01$, $^{***}p < 0.001$, $^{****}p < 0.0001$; ns, not significant ($p > 0.05$).

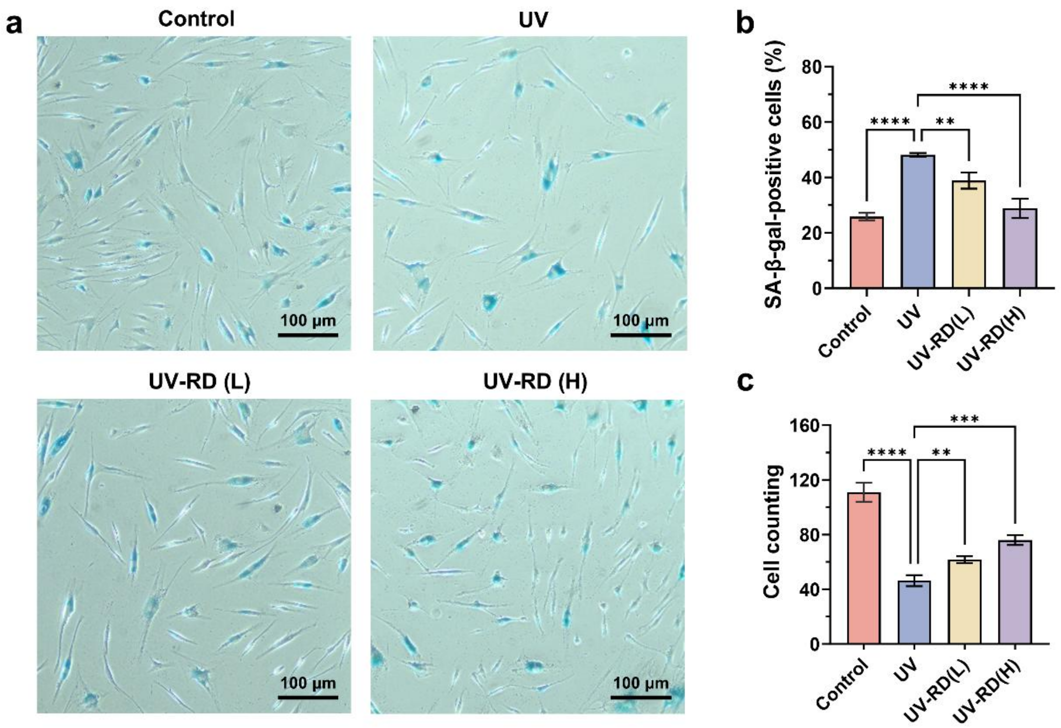

Fig. S4. Effects of RDPM hydrogel on UV-induced cellular senescence in HDFs. a) Representative micrographs showing cellular senescence (marked by SA-β-gal staining in blue) in different treatment groups. Control group, no UV-exposure and no treatment; UV, UV-RD (L) and UV-RD (H) groups: UV-exposure followed by no treatment (0 μg/mL), low-dose *RD* (5 μg/mL), and high-dose *RD* (15 μg /mL) treatments, respectively. b) SA-β-gal positive cell percentages and c) cell counting in the micrographs. Data are presented as mean ± SD (n=3), with comparisons performed by one-way ANOVA. $^{**}p$ <0.01, $^{***}p$ <0.001, and $^{****}p$ < 0.0001.
